# Biodegradable Pectin/Starch-Based Films Applied on Fresh Pears

**DOI:** 10.1021/acsomega.4c08591

**Published:** 2025-06-05

**Authors:** Marina L. C. Chaves, Guilherme A. M. Jesus, Michael C. Castro, Andressa R. S. Bruni, Johny P. Monteiro, Oscar O. SantosJunior, Alessandro F. Martins, Elton G. Bonafé

**Affiliations:** † Laboratory of Materials, Macromolecules, and Composites (LaMMAC), Federal University of Technology—Paraná (UTFPR), Apucarana, Paraná 86812-460, Brazil; ‡ Analytical Apllied in Lipids, Sterols, and Antioxidants (APLE-A), State University of Maringá (UEM), Maringá, Paraná 87020-900, Brazil; § Federal Institute of Paraná (IFPR), Pitanga, Paraná 85200-000, Brazil; ∥ Department of Chemistry, Pittsburgh State University (PSU), Pittsburgh, Kansas 66762, United States

## Abstract

Packaging composed of polysaccharides has emerged as an alternative to petroleum-based commercial products. Thus, this research aimed to develop, optimize, characterize, and apply biodegradable pectin/starch-based films for fresh pears. All film-forming formulations were characterized. Digital images and optical properties suggest stable, transparent, and efficient ultraviolet (UV) blocking films, especially UVC-UVB and partially UVA. Thermal analysis indicated significant mass loss at high temperatures (above 250 °C). The films were permeable to water vapor (0.305–0.255 g × mm/h × m^2^ × kPa) and impermeable to oil. Scanning electron microscopy revealed surface and cross-sectional changes in the material that influenced its mechanical properties, including tensile strength (0.029–0.041 MPa), Young’s modulus (1.42–2.21 MPa), and elongation at break (1.69–2.98%). The films showed a water solubility of around 74% (w/w) with a maximum swelling of 233%. In addition, statistical tools facilitated optimization and data interpretation. The Simplex lattice mixture design indicated that the film containing 100% pectin (Pec-100) was more resistant and less permeable to water vapor, while principal component analysis attributed high transparency and rigidity. In this sense, the Pec-100 material showed potential for film application on fresh pears. Digital images recorded during storage suggested that coated fruits appeared fresh after 15 days. On the other hand, uncoated pears displayed degradation points starting from the tenth day. Therefore, the renewable-source-based film demonstrated promising fruit preservation results.

## Graphical abstract

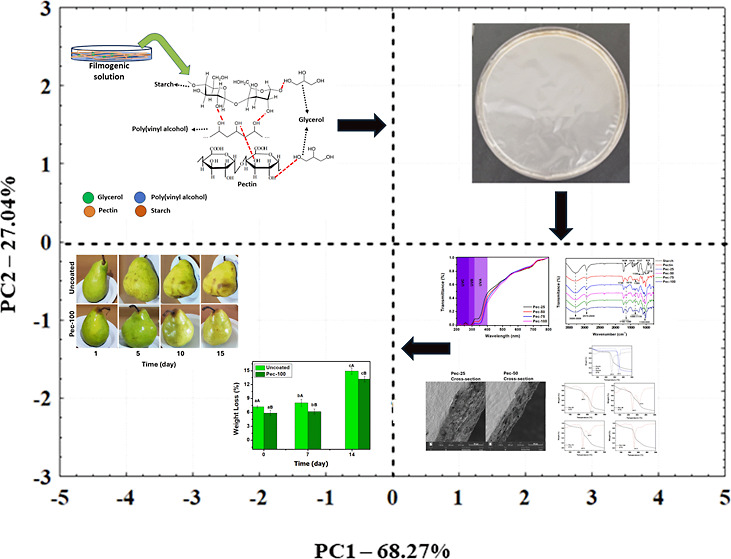

## 1 Introduction

Most food packaging made from petroleum derivatives has serious environmental problems. Materials based on nonrenewable sources exhibit resistance to chemicals, weather conditions, and biodegradation, leading to long-term ecological damage.^1^ Replacing synthetic materials with natural ones is an attractive alternative.^2^ Thus, biodegradable films produced from starch are gaining prominence.^3^ Starch offers several advantages: being inexpensive, abundant, renewable, and biodegradable. Additionally, due to its film-forming properties, starch films can be processed by the casting method, resulting in transparent, odorless, biocompatible, and environmentally friendly materials.^1,4,5^

However, biodegradable packaging composed solely of starch exhibits limited mechanical, barrier properties, and interfacial adhesion. Blending with other polysaccharides has been a promising strategy to overcome this.^6–9^ Considering the low cost, availability, biocompatibility, nontoxic, and excellent film-forming properties, pectin becomes an excellent alternative. Butler et al. reported that cellulose and chitosan were subjects extensively investigated while pectin remains underutilized. The authors also suggest that pectin extraction from food byproducts, such as citrus peels (waste generated in tons annually), contributes to the circular economy and promotes sustainability.^10^ Additionally, pectin is generally recognized as safe for food industry,^11^ employed as a stabilizer, emulsifier, thickener, and gelling agent,^12^ and cosmetics.^13^ In this context, this study explores, for the first time, the development of a biodegradable film from commercial polysaccharides donated by the food industry with potential applications in food packaging.

Blending starch and the negatively charged carboxylate groups of pectin promote high interfacial adhesion, improving the mechanical properties of the material.^14^ In addition to polysaccharide mixtures, plasticizers also improve the elasticity of the materials. They are generally composed of nonvolatile, low-molecular-weight polyols, with glycerol highlighted.^2^ Furthermore, reinforcing materials, such as poly­(vinyl alcohol) (PVA), have also been employed. PVA is a biodegradable synthetic polymer that is easy to process and has excellent properties (film-forming, mechanical, and barrier) and transparency.^15^

Therefore, blends involving starch/pectin plasticized with glycerol and reinforced with PVA are promising to produce films for minimally processed fruit applications. According to Dilucia and coauthors, the postharvest period and transportation of climacteric crops, such as pears, result in significant losses. Pears are sensitive to adverse conditions during transport and storage.^16^ Thus, films are an alternative to reduce waste during this step.^17^

Then, this study aimed to optimize the pectin/starch ratio using the Simplex lattice multivariate statistical method employing commercial polysaccharides used as food ingredients. The multivariate technique assists in obtaining materials with the desired properties by the optimal pectin/starch ratio. The material selected from the optimized conditions was applied as a film on fresh pears and monitored for 15 days in a climatized room. Before application, all formulations were characterized by Fourier transform infrared spectroscopy (FTIR), scanning electron microscopy (SEM), thermogravimetric analysis (TGA), mechanical properties (tensile strength, elongation at break, and Young’s modulus), optical measurements (color, opacity, and UV-blocking), water vapor and oil permeability, moisture content, water solubility, and swelling. Also, principal component analysis (PCA) helped to interpret the numerous results obtained from characterization. Thus, to the best of our knowledge, the use of multivariate statistical techniques in the optimization (Simplex lattice design) and data interpretation (PCA) for preparing pectin- and starch-based biodegradable films is underexplored.

## 2 Materials and Methods

### 2.1 Materials

The films were produced from citrus pectin with an esterification degree of 56% (CPKelco, Brazil) and corn starch (25–28% amylose content) (Indemil company and Comércio S/A (Brazil). PVA (BASF, Germany) and glycerol were purchased from Dinâmica (Brazil).

### 2.2 Preparation of the Pectin/Starch Films

The pectin/starch films were produced from the casting method, as described earlier.^18,19^ The pectin/starch ratios were optimized for tensile strength and water vapor permeability responses by using a Simplex lattice mixture design. The total mixture limit of pectin/starch was 3 g (100%). Table 1 displays the concentrations of pectin and starch mixtures ranging from 0 to 100% (0–3 g).

**Table 1:** Composition and Simplex Lattice Design of the Films^a^

films	glycerol (g/%, w/v)	PVA (g/%,w/v)	pectin (g/%, w/v)	starch (g/%,w/v)
Pec-0	0.75/0.75	0.75/0.75	0/0	3.0/3
Pec-25	0.75/0.75	0.75/0.75	0.75/0.75	2.25/2.25
Pec-50	0.75/0.75	0.75/0.75	1.5/1.5	1.5/1.5
Pec-75	0.75/0.75	0.75/0.75	2.25/2.25	0.75/0.75
Pec-100	0.75/0.75	0.75/0.75	3.0/3	-/0

a The weight content (%, w/w) of pectin/starch/PVA solutions is expressed in % w/v in 100 mL of solution. PVA: poly­(vinyl alcohol). The films were developed in triplicate.

### 2.3 Characterization of the Pectin/Starch Films

The interaction between the different functional groups was estimated using Fourier transform infrared spectroscopy in the attenuated total reflectance mode (FTIR-ATR), a Nicolet iN10 model equipped with a zinc selenide (ZnSe) crystal. The spectra were obtained in the spectral range of 4.000 to 400 cm^–1^, with a resolution of 4 cm^–1^ and 64 scans.

Thermogravimetric analysis (TGA) was carried out using a thermogravimetric analyzer (Shimadzu, TGA-50, Japan). The thermograms were recorded under an argon flow of 50 mL/min, heated from 25 to 800 °C at 10 °C/min.

The micrographs of the surfaces and cross section of pectin/starch films were carried out using scanning electron microscopy (SEM) with a 3 kV accelerating voltage (TESCAN VEGA, Tescan). The samples were placed on the stub, coated with a thin gold layer, and stored in a desiccator for SEM analysis.

Film thickness measurements were carried out using an electronic digital micrometer (YST tech, model YUANLS-H4024, China). The averages are the result of 15 random readings for each sample. The mechanical properties of the films were tested by tensile strength (σ, MPa), elongation at break (ε, %), and Young’s modulus (∑, MPa). The 6 × 0.5 specimens (cut in six repetitions) were measured using a TA-XTplus texture analyzer (Stable Micro Systems, Surrey, England) in the tensile mode according to ASTM D882-02,^19^ with modifications. The tweezers, one fixed to the immobile base and the other moving at a speed of 2 mm × s^–1^, covered a maximum distance of 100 mm.

The films’ water vapor permeability (WVP) was estimated as previously reported^20^ with modifications. Initially, the films were conditioned in a desiccator at 25 °C under controlled relative humidity (RH) at 53% with calcium chloride for 72 h. The films were then attached to the top of test capsules containing magnesium chloride (RH 33%), weighed *W*_0_, transferred to a desiccator containing sodium chloride (RH 75%), and kept for 72 h at ±2% RH. The test capsules were weighed at different intervals until they reached a constant permeability rate (*w*/t). The WVP was obtained by eq 1

1 $$WVP=\frac{W\times L}{A\times t\times \Delta P}$$

where *W* is the water weight permeated through the film (g), *L* is the film thickness (mm), *A* is the permeation area (m^2^), *t* is the permeation time (h), and Δ*P* is the water vapor pressure (Pa) difference in the permeation capsules related to the interior and exterior environment.

The oil permeability through the films was estimated as described before.^21^ Film cuts (2 × 2 cm) were repositioned to the nozzle of a glass vial containing 5 mL of commercial soybean oil. Then, the flask was turned over onto the surface of a preweighed filter for 48 h at 25 °C. The filter paper was reweighed and provides the mass of oil permeated through the material. Oil permeability was determined by eq 2

2 $$oil\;permeability=\frac{\Delta W\times X}{A\times t}$$

where Δ*W* describes the mass of oil permeated (g), *X* is the film thickness (mm), *A* is the exposure area (m^2^), and *t* is the analysis time (days).

### 2.4 Moisture Content, Water Solubility, Swelling Degree, and Swelling Kinetics

The moisture content was determined according to the methodology proposed by Bruni and coauthors.^15^ Specimens with dimensions of 2 × 2 cm were initially weighed (*W*_O_) and relocated to an oven at 103 ± 2 °C until constant weight (*W*_f_). Then, the moisture content (%, w/w) was acquired by eq 3

3 $$moisture\;content\;\left(\%\right)=\frac{\left(W_{O}-W_{f}\right)}{W_{O}}\times 100$$

where *W*_O_ and *W*_f_ corresponds to the initial and final weights of the dried films, respectively.

The water solubility of the film was determined using gravimetric measurements, as previously reported.^15^ The dried and previously weighed films *W*_0_ were transferred to Falcon tubes holding 30 mL of distilled water and maintained for 24 h at 25 °C. After the interval, the solution was filtered through preweighed filter paper. The filter was then dried in an oven at 103 ± 2 °C for 24 h and weighed again to acquire the final mass *W*_f_. The water solubility (% by weight) was determined using eq 4

4 $$water\;solubility=\frac{\left(W_{0}-W_{f}\right)}{W_{f}}\times 100$$

where *W*_0_ and *W*_f_ are the initial and nondissolved film weights, respectively.

The swelling degree was performed according to a protocol published before.^15^ The dry film (2 × 2 cm) was previously weighed (*W*_0_) and inserted into Falcon tubes (100 mL) with 30 mL of distilled water. After that, the tubes were stirred at 100 rpm for 24 h at room temperature. Then, the weight gain after 24 h provided the final weight (*W*_f_). The swelling degree values were calculated using eq 5

5 $$swelling\;degree\;\left(\%\right)=\frac{\left(W_{f}-W_{0}\right)}{W_{0}}\times 100$$

where *W*_f_ and *W*_0_ are the weight of the films after and before swelling, respectively.

The kinetic study of film swelling was estimated by measuring the mass gain at different time intervals (1, 10, 30, 60, 120, 180, 300, and 600 min). The mass gain (g) versus time (min) curve provides the kinetic profile of the films. Each point was calculated using eq 5.

### 2.5 Optical Properties

The apparent opacity and UV-blocking of the films was evaluated as described by Bruni and coauthors.^15^ The film cuts (1 × 4 cm) were transferred to a quartz cuvette (1 × 5 cm) and analyzed in triplicate at 200–800 nm using a Thermo Fisher Scientific spectrophotometer (Finland) model Genesys 10-S. The UV barrier (180–380 nm) was measured in the transmittance mode, while the apparent opacity was measured in absorbance (550 nm). Apparent opacity was calculated using eq 6

6 $$apparent\;opacity=\frac{{Abs}_{550}}{X}$$

where Abs_550_ is the absorbance at 550 nm and *X* is the film thickness (μm).

The brightness (L*), red/green (a*), and yellow/blue (b*) color parameters were estimated in triplicate by using a Konica Minolta CR-400 digital colorimeter (Japan), previously calibrated according to the manufacturer. The measurements were presented according to the CIE (Commission Internationale de l’Eclairage) system. The color difference (Δ*E*) was calculated using eq 7

7 $$\Delta E=\sqrt{{\left(L^{*}-L\right)}^{2}+{\left(a^{*}-a\right)}^{2}+{\left(b^{*}-b\right)}^{2}}$$

where L* (93.83), a* (−0.67), and b* (4.00) refers to the color parameters of the white plate.

### 2.6 Film Performance on Fresh Pears

The film was applied to fresh pears obtained from the local market in Maringá, PR, Brazil (23° 25′ 31″ S/51° 56′ 19″ W), according to a previous study.^17^ The classified pears (based on size, color, shape, and physical condition) were disinfected by immersion in a sodium hypochlorite solution (0.1 g/L) for 10 min. After rinsing with water and drying, the fruits were immersed in the film-forming solution (Pec-100) for approximately 30 s and then transferred to plastic trays. The coated and uncoated (control) pears were stored in a climate-controlled environment (23 ± 2 °C and 78% RH). The coated fruits were monitored over 15 days by evaluating the weight loss and capturing digital images at different intervals (0, 5, 10, and 15 days). The weight loss was calculated in accordance with eq 8

8 $$weight\;loss\;\left(\%\right)=\frac{\left(W_{0}-W_{f}\right)}{W_{0}}\times 100$$

where *W*_0_ and *W*_f_ are the initial and final weight of the films.

### 2.7 Statistical Analysis

The results obtained were statistically analyzed by variance (ANOVA) and Tukey test at 5% significance using software PAST (Statsoft Inc. Brazil). PCA was performed to identify differences or similarities among the data obtained from the various measurements. The different analysis results were standardized between 0 and 1, avoiding biases based on individual magnitudes.^22^

## 3 Results and Discussion

### 3.1 Preparation of the Pectin/Starch Films

Biodegradable films based on pectin and starch were produced by using different formulations (Table 1). Previous experiments have indicated that films comprising exclusively pectin or starch, exhibited fragility and low mechanical strength. The diminished stability of these formulations can be ascribed to the three-dimensional structure of the polysaccharides. Starch macromolecules consist of amylose and amylopectin. Amylose, forming the linear portion, facilitates the self-assembly.^19^ Conversely, amylopectin, characterized by its branched structure, leads to decreased linearity, thereby hindering interchain interactions.^23^

Due to its complex molecular structure, pectin lacks a fully defined chemical conformation. The macromolecule contains α- *d* -galacturonic acid residues (1 → 4) branched with diverse sugars. These can be further delineated into anionic linear domains devoid of side groups and nonanionic branched regions.^24^ The anionic groups, stemming from the deprotonation of carboxylic acids (−COOH, pKa 2.9–3.2) in aqueous media (pH = 7.0), induce electrostatic repulsion among the pectin molecules.^25^ This fact justifies the diminished stability observed in this system.

Thus, glycerol (plasticizer) and PVA (reinforcing agent) were employed to enhance the mechanical and barrier properties of the films. Both agents can link by H-bonds with polysaccharide −OH groups. This intermolecular cross-linking among pectin–glycerol, PVA–pectin, starch–glycerol, starch–PVA, starch–glycerol–pectin, and starch–PVA–pectin imparted significant reinforcement to the film matrix (Scheme 2). Prior investigations have demonstrated that the incorporation of glycerol (0.75% w/v) and PVA (0.75% w/v) into biodegradable films based on pectin, κ-carrageenan, and cassava starch yielded materials with improved mechanical properties.^15,19,26^

**Scheme 1:**
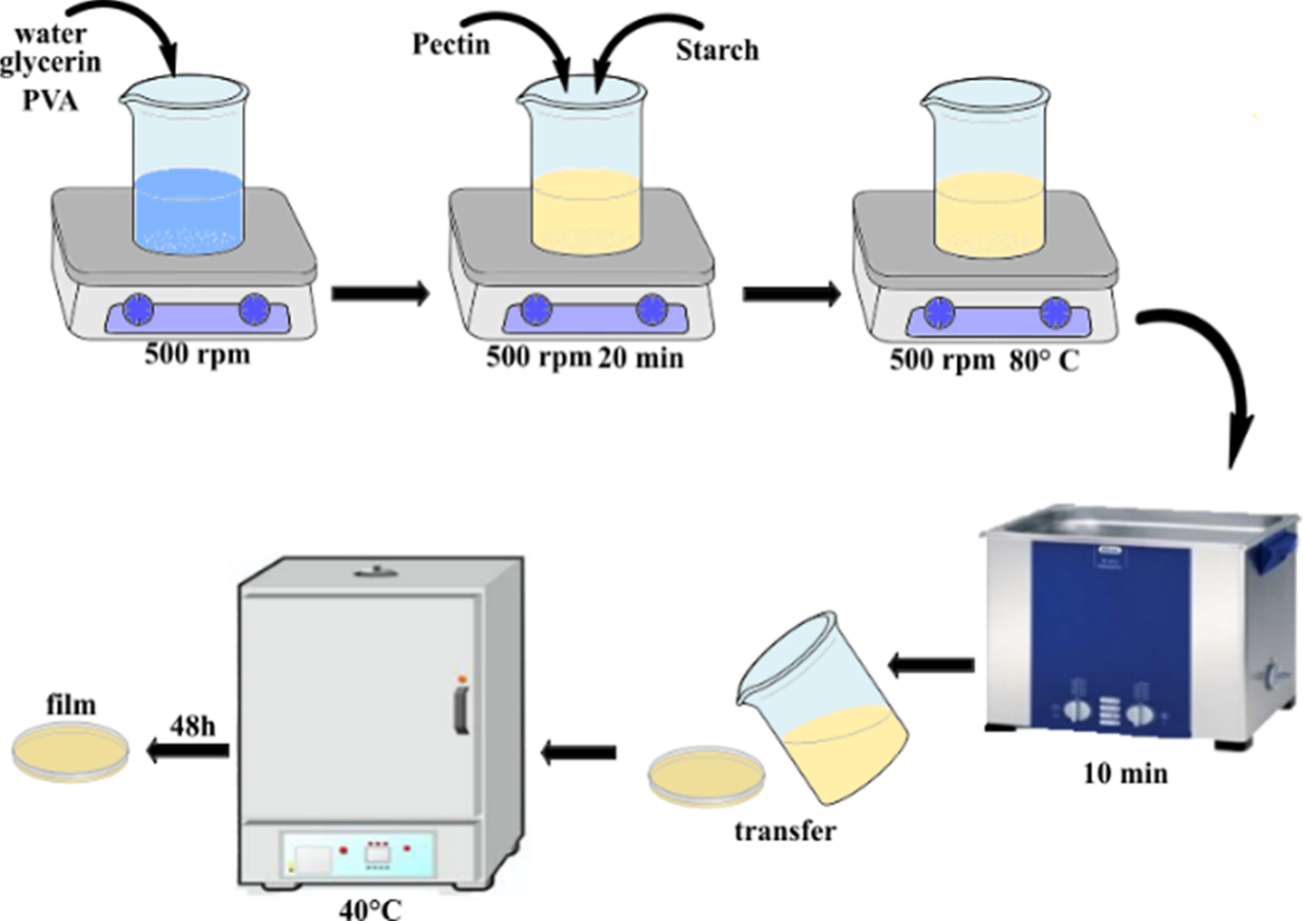
Preparation Steps of the Pectin/Starch Films

**Scheme 2:**
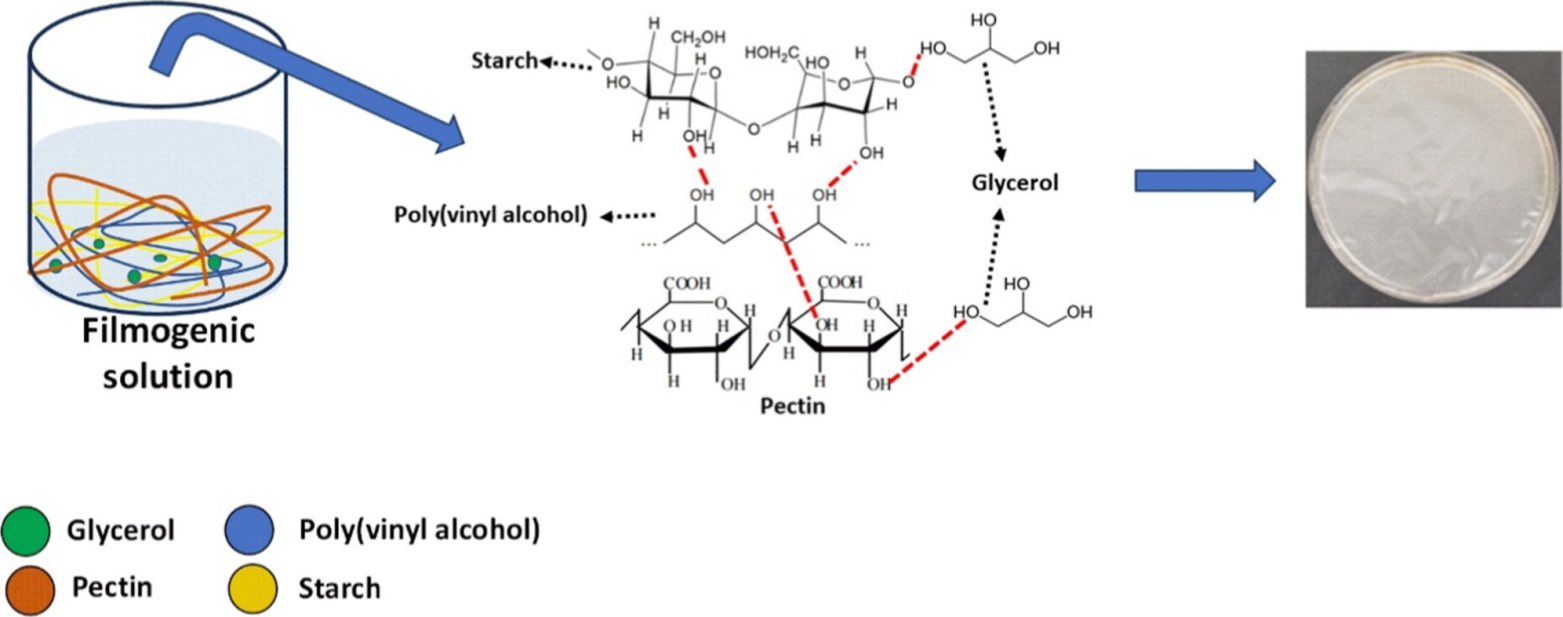
Intermolecular Interactions among Filmogenic Solution Precursors

The digital images of the produced formulations are presented in Figure 1, except for the Pec-0. Visually, the materials exhibited transparency, homogeneity, processability, and ease of handling, except for the Pec-0. The mixture containing only starch resulted in a brittle material that was difficult to demold. The high starch content in the mixture intensifies the intermolecular interactions of the polymer network, especially the H-bonds between the −OH groups. These strong interactions compromise the mobility between the chains, producing a rigid and low-flexible material. These features eliminated the pec-0 from further characterizations. On the other hand, adding pectin to the formulations resulted in improved materials. In this regard, characterizations were performed on the Pec-25, Pec-50, Pec-75, and Pec-100.

**Figure 1:**
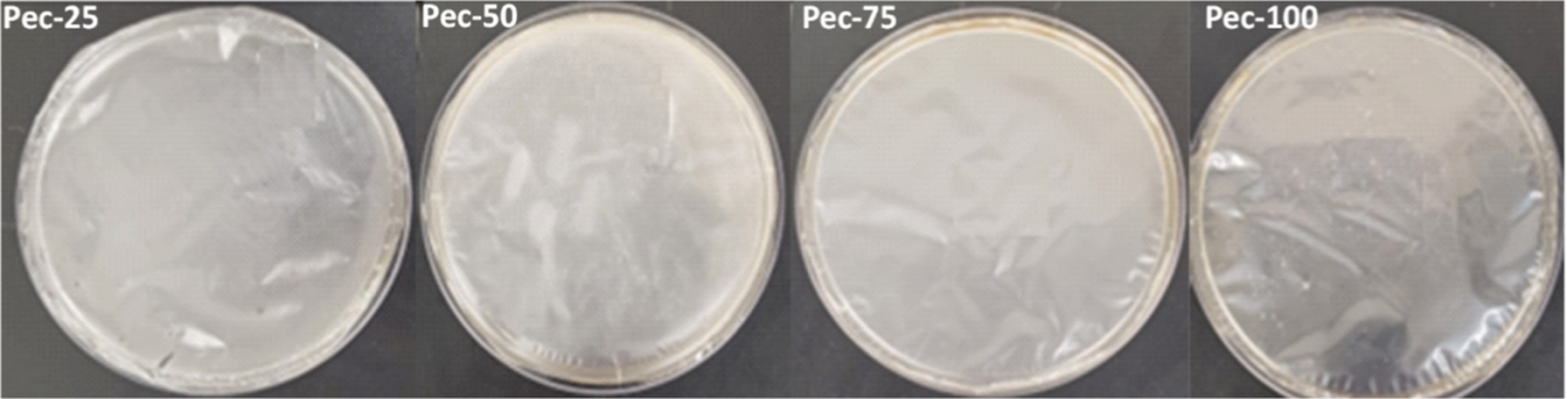
Digital images of films based on pectin/starch.

### 3.2 Fourier Transform Infrared Spectroscopy

Figure 2 displays the FTIR spectra for pectin, starch, and films. The spectra exhibit a broad band between 3500 and 3000 cm^–1^ associated with stretching −OH groups from hydroxyl sites in starch and pectin macromolecules. Signals between 2970 and 2850 cm^–1^ and 1100 and 1018 cm^–1^ can be attributed to the symmetric and asymmetric stretching of −CH_2_ and −CH_3,_ and C–O–C bonds, respectively.^26^ Additionally, the FTIR spectrum of starch shows a band related to water molecules at 1639 cm^–1^, C–H deformation at 1410 cm^–1^, C–OH stretching at 1337 cm^–1^, and stretching of C–O–C bonds at 925 cm^–1^, attributed to the repeating unit of 3,6-dehydrated galactose.^19,27^ On the other hand, signals between 1740 and 1610 cm^–1^ and 1385 cm^–1^ are attributed to ester and carboxylic acid and C–H from *O*-esterified groups from pectin.^25^ The same signals identified in the original FTIR spectra of starch and pectin are also detected in the spectra of the filmogenic mixtures. However, the intermolecular interactions, mainly hydrogen bonding, among the polymers, plasticizer, and water broaden and shift the maximum absorption characteristic bands. The H-bond formed among them is clarified in Scheme 2. In addition, the signals at 1760–1560 cm^–1^ and 1490–1170 cm^–1^, missing in the precursors, justify these findings. These bands can be attributed to the overlap of ester and carboxylate groups of pectin and −CH, −COH groups of starch.^26^

**Figure 2:**
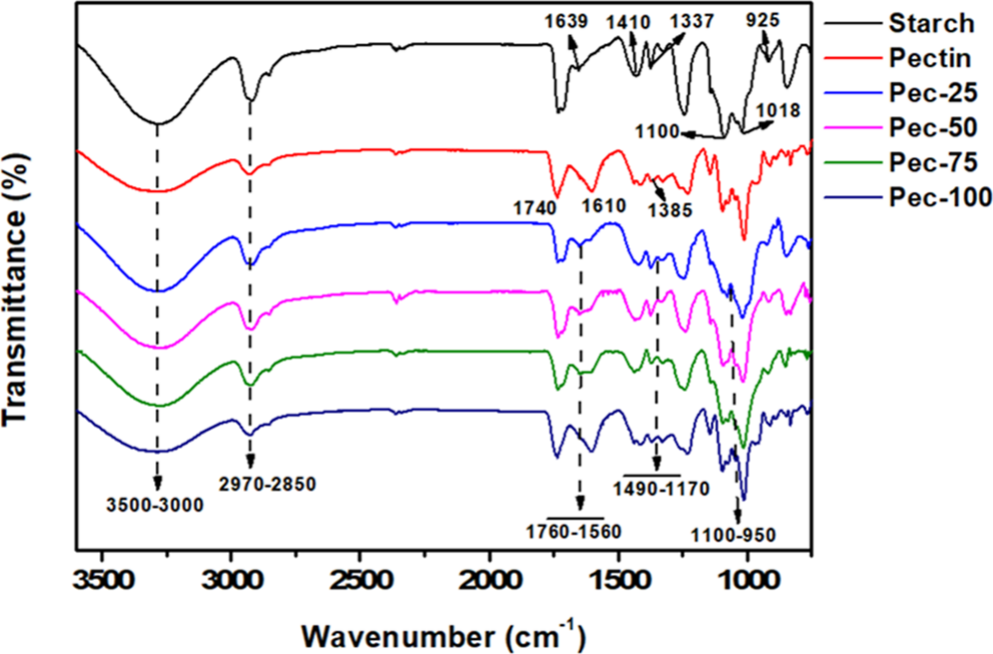
FTIR spectra of the films.

### 3.3 Thermogravimetric Analysis

The thermal behavior of the pectin/starch films evaluated by thermogravimetric analysis (TGA) is shown in Figure 3. The curves indicate mass loss occurring in the three regions. The first region corresponds to the loss of water and volatile compounds, which tend to evaporate at temperatures up to 150 °C. The second and third regions are associated with the degradation of glycerol and polymeric chains, occurring between 200 and 350 °C, and the oxidation and degradation of byproducts at temperatures above 350 °C.^17,28^

**Figure 3:**
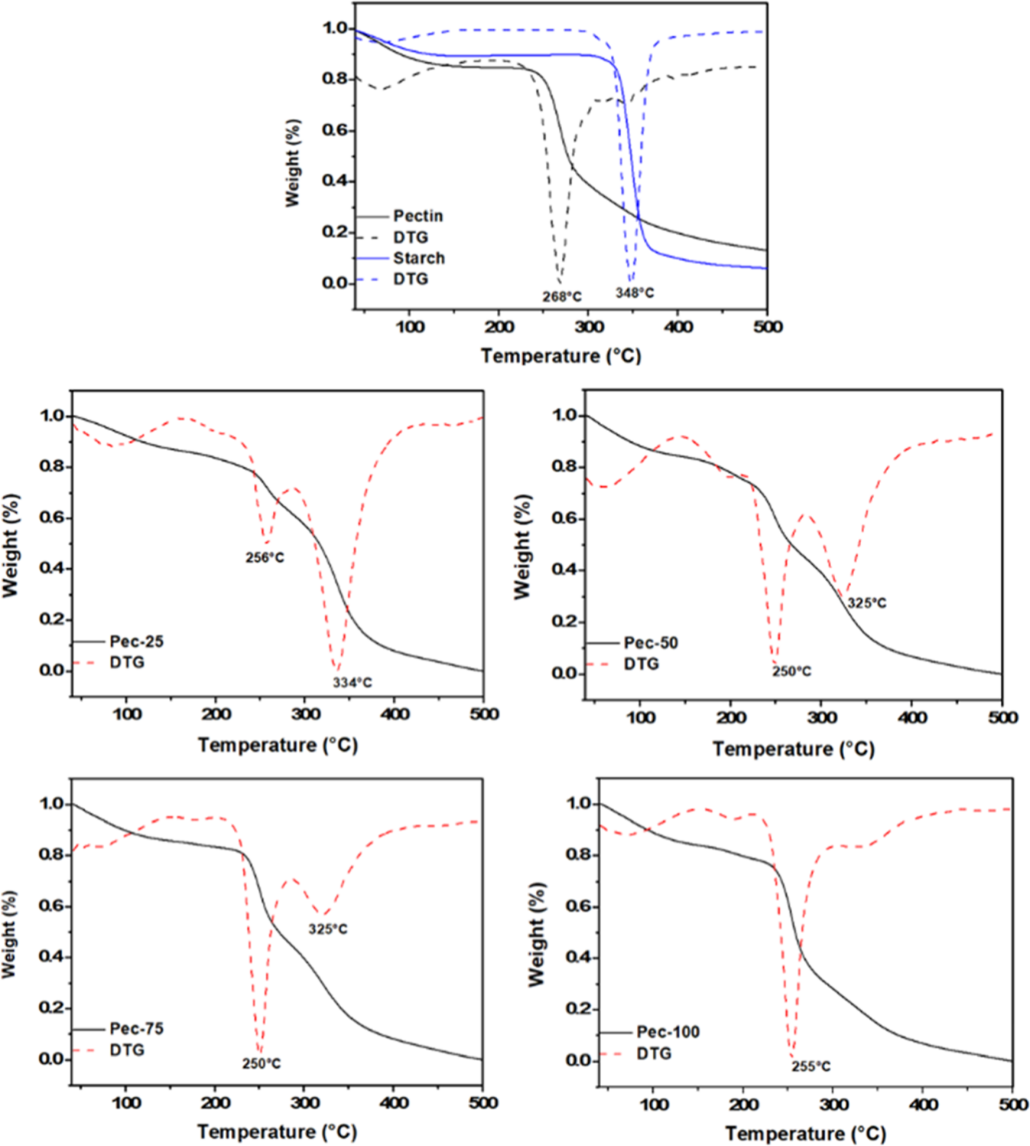
TGA curves of the precursors and films.

Additionally, the TG/DTG curves of the films exhibit two intense signals corresponding to major degradation events. They occur between 250–256 °C and 325–334 °C, accounting for approximately 70% of the total mass loss. However, the thermal profile of the signals varies, depending on polysaccharide composition. For example, while the material containing a 50:50 (pectin/starch) ratio exhibits comparable mass loss, the Pec-75 (75% pectin) and Pec-25 (75% starch) show a higher mass loss, all of them at the same signals between 250–256 °C and 325–334 °C. The DTG of the original starch and pectin before the mixture supports this. Pectin and starch exhibit the same individual signals at 348 and 268 °C, respectively.

Furthermore, the increase in the pectin content in the mixture reduced the degradation temperature in Pec-50 and Pec-75 films. A similar behavior has been previously reported.^29^ Pectin is a branched polysaccharide. Its side chains create steric hindrance with neighboring molecules, increasing the intermolecular spacing. This effect reduces molecule-to-molecule interactions, leading to lower degradation temperatures.^25,29^ Thus, the variation in temperature for each event is associated with the breaking and formation of new interactions.^30^ Different degradation temperatures of the precursors compared to starch/κ-carrageenan composite films have also been reported in a previous study.^19^ Pectin/starch-based composite films loaded with eggshell microparticles exhibited similar behavior. Adding microparticles to the material disrupted the pectin/starch system, reducing the maximum degradation temperature.^31^

### 3.4 Scanning Electron Microscopy

Figure 4 shows the scanning electron microscopy (SEM) micrographs of the surface and fractures of pectin/starch films at different proportions. The images suggest an irregular, wavy surface with no visible cracks. The cross section also does not reveal severe ruptures in the material but indicates the presence of pores, especially in the Pec-25 and Pec-50 films. Therefore, the film composition reflects changes in the surface and cross section, which are correlated with other material properties (mechanical and barrier characteristics).^32^ The cross-sectional images show that films with a higher pectin content (Pec-75 and Pec-100) tend to be more compact. The mechanical parameters and the gas permeability barrier (discussed in Section 3.5) further illustrate these findings. Pectin-rich films exhibit greater stiffness and lower water vapor permeability. Variations in gas permeability measurements and tensile strength were correlated to SEM images of pectin/PVA composite films plasticized with 35% (w/w polymer) glycerol. The authors attribute these changes to intermolecular interactions, which modify the materials’ physical properties.^33,34^

**Figure 4:**
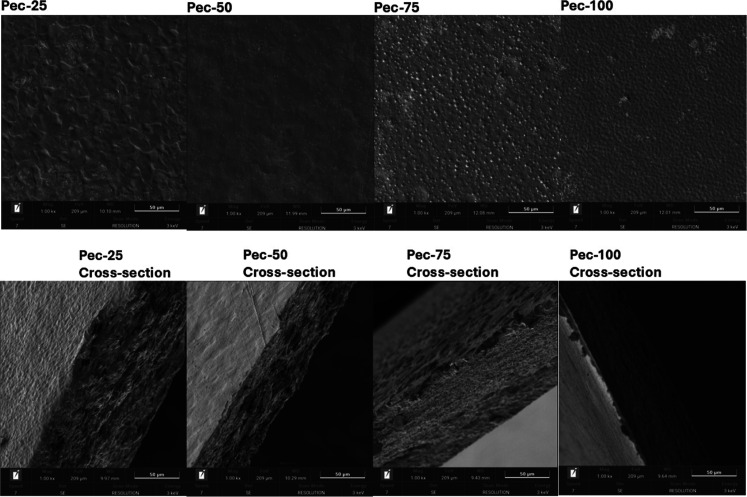
SEM images of the pectin/starch films. Scale 50 μm, magnification 1000×, and 3000 eV.

### 3.5 Thickness, Mechanical Properties, Water Vapor, and Oil Permeation

The thickness measurements of the films, used to calculate the mechanical properties, ranged from 65 to 82 μm (*p* ≤ 0.05). These properties were estimated on the tensile strength (σ—MPa), rupture stress (ε—%), and Young’s modulus (*E*—MPa) parameters extracted from stress–strain curves (Figure 5 and Table 2). The tensile strength measurements showed no significant difference for the Pec-25, Pec-50, and Pec-75 materials (*p* ≤ 0.05). However, increased pectin in the filmogenic mixture led to a gradual increase in σ, reflected in Young’s modulus. Pec-100 was responsible for the highest *E*-value. Thus, a lower starch content delivers stiffer materials. Biodegradable films based on κ-carrageenan/starch plasticized with 20% glycerol (w/v) reached similar conclusions. The rich mixture in κ-carrageenan produced rigid films.^19^ Contrarily, resistant materials often exhibit less flexibility. The elongation measurement and swelling degree support these findings because the Pec-100 showed lower flexibility and ability to expand the polymer network.

**Figure 5:**
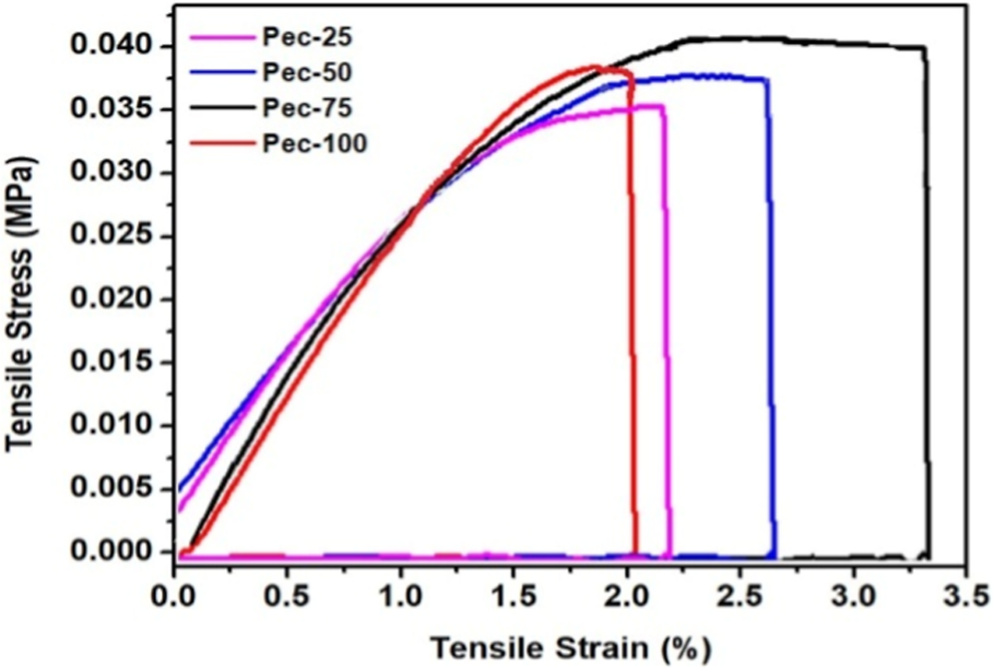
Representative stress–strain curves of the films.

**Table 2:** Mechanical Properties and WVP Measurements of the Films^a^

films	*E*	Σ	Ε	WVP	oil permeability
Pec-25	1.42 ± 0.5^b^	0.029 ± 0.008^b^	1.69 ± 0.56^a^	0.305 ± 0.30^b^	Nd
Pec-50	1.88 ± 0.1^a^	0.033 ± 0.004^b^	2.0 ± 0.64^c^	0.301 ± 0.25^b^	Nd
Pec-75	2.21 ± 0.1^a^	0.035 ± 0.009^b^	2.98 ± 0.35^b^	0.293 ± 0.27^b^	Nd
Pec-100	2.19 ± 0.2^a^	0.041 ± 0.003^a^	1.58 ± 0.36^a^	0.255 ± 0.60^a^	Nd

a Results are presented as mean ± standard deviation. Different letters in the same column indicate significant differences (*p* ≤ 0.05) according to Tukey’s test. ***E*** —Young’s modulus (MPa); σ—tensile strength (MPa); ε—elongation at the break (%); WVP—water vapor permeation; and Pec—pectin. Nd—not detected.

High starch content in the blend assembles unstable films (discussed in Section 3.1). The elevated levels of hydroxyl groups enhance the intermolecular interactions of the polysaccharide chains. The proximity of macromolecules results in brittle materials of low flexibility, even when plasticized with glycerol. Adding pectin into the blend assists in breaking the crystallinity between starch molecules due to the intermolecular rupture and formation of new H-bonds between starch–starch and starch-pectin, respectively.^35^

Starch (5% w/w)/pectin (5% w/w) composite films with a 75/25 (starch/pectin) ratio loaded with eggshells at 0–8% (w/w polymer) exhibited thicknesses ranging from 28 to 36 μm. The authors reported composite materials with a higher tensile strength (σ, 15–19 MPa) and stiffness (*E*, 2.2–2.8 GPa) but lower flexibility (ε, 1.9–2.4%). The results indicate that adding eggshells contributes to these improvements.^31^ Pectin-based (1% w/w)/PVA (0.05% w/w) films plasticized with glycerol and activated with sporopollenin (0–0.2% w/w) showed thicknesses between 120 and 250 μm. Composite films delivered σ of 4.39–7.44 MPa and ε of 10–37%.^30^ Zuzanna and coauthors produced starch-based films loaded with nanosilica to evaluate the effect of hydrophobicity on the material surfaces. The films were plasticized with glycerol, 40–80% w/w (concerning starch mass), and loaded with nanosilica (2.5–5.0% w/w, concerning starch). The materials yielded a tensile strength of 1–5 MPa, an elongation at break of 29–116%, and Young’s modulus of 5–94 MPa.^5^ A previous study reported the development of starch-based films (4%, w/v) enriched with microcrystalline cellulose (5–15%, w/w starch mass) and plasticized with glycerol at 12% w/w of starch. The tensile strength results ranged from 1.5 to 5 MPa, elongation at break from 40% to 90%, and Young’s modulus from 50 to 250 MPa.^36^

The measurements of WVP are presented in Table 5. The values ranged from 0.255 to 0.305 g × mm/h × m^2^ × kPa. The Pec-25, Pec-50, and Pec-75 films exhibited identical WVP according to the Tukey test (*p* ≤ 0.05). The results suggest that WVP values decrease with the starch reduction in the blend. The strong interaction between water and starch molecules, especially by H-bonds, may justify these findings. A previous study reported that additives prone to interacting via H-bonds with water increased WVP in starch-based films. In the research, higher levels of sericin in the filmogenic starch/sericin combinations resulted in higher WVP.^27^

Additionally, the mechanical parameters (σ and Σ) agree with the results obtained, where the more compact and rigid materials exhibited lower WVP. Compact and dense polymeric networks generally exhibit fewer free spaces between the chains, which hinders vapor permeation. Diksha Lingait and collaborators report that dense polymeric networks impede water vapor exchange, making the material less permeable.^30^ Jesus and coworkers published similar results. The researchers developed active biodegradable films based on κ-carrageenan (2.5 and 3% w/w) enriched with gallic acid (6.25 and 10% w/w of polysaccharide). The blends exhibited results ranging from 3.75 × 10^–12^ to 6.33 × 10^–12^ g × mm/h × m^2^ × kPa. The lower permeation rates were related to the more resistant materials.^22^ Similar trends have also been reported previously.^37^ Starch/carboxymethylcellulose-based films (0.5–2.0% w/w) plasticized with glycerol at 40% w/w (starch weight) yielded values ranging from 1.668 to 2.948 g × mm/d × m^2^ × kPa.^37^ Chambi and Grosso also found values close to those obtained here. They reported WVP values ranging from 0.249 to 0.345 g × mm/d × m^2^ × kPa in methylcellulose/glucomannan/pectin/gelatin films.^38^

In contrast, all materials resisted the oil permeation test for 48 h. The hydrophobic nature of the oil hinders its permeation through the hydrophilic films (water solubility >70% (discussed in Section 3.7) support this. Farhan and Hani produced edible films composed of κ-carrageenan 2% w/w plasticized with glycerol or sorbitol at 20, 25, and 30% w/w impermeable to oil passage.^39^

### 3.6 Optimization of the Pectin/Starch Ratio for Preparing Biodegradable Films

The optimization was performed by using the Simplex lattice mixture design model. The data for tensile strength (σ) and water vapor permeation (WVP) fit linear and quadratic order models, respectively. Table 3 presents the analysis of variance (ANOVA) for the models. The high *F*-values (38.72) and *p*-values < 0.05 indicate the significance of the linear model and the linear component of the mixture. Other evaluative parameters of the model, such as *R*-squared, predicted *R*-squared, adjusted *R*-squared, and adequate precision, also support the previous findings. The *R*-squared value > 0.9 suggests a high correlation between the experimental and predicted values by the model; the predicted *R*-squared of 0.7169 is consistent with the adjusted *R*-squared of 0.8829; a signal/noise ratio (adequate precision) > 4 enables the model to navigate the design space effectively (Statsoft, 2005). These same parameters confirm the significance of the quadratic model used for the WVP response.

**Table 3:** Analysis of Variance (ANOVA) of the Simplex Lattice Mixture Design^a^

	tensile strength (σ)			
source	sum of squares	mean square	*F*-values	*p*-values
linear model	0.73	0.73	38.72	**0.0034**
linear mixture	0.73	0.73	38.72	**0.0034**
residual	0.075	0.019		
cor total	0.80			
*R*-squared	0.9064			
adj *R*-squared	0.8829			
pred *R*-squared	0.7169			
adeq precision	11.806			

WVP				
quadratic model	1.17	0.58	29.89	**0.0104**
linear mixture	0.032	0.032	1.64	0.2906
starch × pectin	1.14	1.14	58.15	**0.0047**
residual	0.059	0.020		
cor total	1.23	0.58		
*R*-squared	0.9522			
adj *R*-squared	0.9204			
pred *R*-squared	0.8185			
adeq precision	10.956			

a Predicted *R*-squared: adj *R*-squared; adjusted *R*-squared: adj *R*-squared; and adequated precision: adeq precision.

Predictions of outcomes from statistical models can be calculated using eq 9 (tensile strength) and eq 10 (WVP). Equation 9 relates positive terms (+0.85000 × Pectin) and negative terms (−0.083333 × Starch). The negative sign accompanying the starch variable indicates a reduction in the material’s tensile strength, while the positive sign suggests an increase. The interpretation of the equation aligns with the experimental measurements. The most resistant material is composed only of pectin in the mixture (Pec-100).

9 tensilestrength=−0083333×starch+085000×pectin

Equation 10 describes the material’s behavior to water vapor permeability response. The model also relates positive (+0.036970 × starch; +4.19879 × starch × pectin) and negative terms (−0.029455 × pectin). In this case, the variables starch and pectin have opposite signs compared with the previous model. Increasing the levels of pectin in the mixture decreased the WVP. The most permeable material to water vapor is produced with a high starch content. The experimental measurements agree with those of the theoretical model. Thus, consistency between the experimental and theoretical data validates the proposed models.

10 WVP=+0036970×starch−0029455×pectin+419879×starch×pectin

### 3.7 Moisture, Water Solubility, Swelling Degree, and Swelling Kinetics

According to Tukey’s test, the moisture content shows no significant difference in the films (*p* ≤ 0.05). Table 4 displays measurements ranging from 10.26 to 11.92%. The essay evaluates the release of volatile compounds, especially water, at 105 °C until a constant mass. Water is maintained in different materials by intermolecular interactions. H-bonds between water and pectin, starch, PVA, and glycerol can be formed through the −OH groups in all precursors. Film-forming mixtures based on starch (100, 80, and 70% w/w)/PVA (0, 10, 20, and 30% w/w) incorporating glycerol (37.5% w/w) and citric acid (1% w/w) exhibited a moisture content between 18 and 24%.^4^ Films composed of carrageenan 2% w/w, plasticized with sorbitol, and enriched with germinated fenugreek seed extract, showed a moisture content of 10–25%.^41^ Farhan and Hani reported a moisture content ranging from 8.8 to 14.5% in κ-carrageenan films using glycerol at different concentrations (20–30%, w/w).^39^

**Table 4:** Moisture and Water Solubility of the Films^a^

films	moisture	water solubility (%)
Pec-100	11.45 ± 0.89^a^	74.27 ± 0.40^b^
Pec-75	11.92 ± 1.27^a^	74.47 ± 0.25^ab^
Pec-50	11.46 ± 0.99^a^	74.95 ± 0.27^ab^
Pec-25	10.26 ± 0.66^a^	75.20 ± 0.40^a^

a Results are presented as mean ± standard deviation. Different letters in the same column indicate significant differences (*p* ≤ 0.05) according to Tukey’s test. Pec: pectin.

The water solubility results demonstrated a notable attraction between the material and the solvent. All materials exhibited measurements above 70% (Table 4). The high affinity for the aqueous medium indicates the hydrophilic characteristics of the material. Statistical testing indicated differences between Pec-100 and Pec-25. The higher solubility of Pec-25 can be justified by the amount of starch in the mixture. Starch consists of more −OH groups in its repeating units than pectin, which interacts strongly with the solvent, explaining the findings. Studies of this nature assist in evaluating the hydrophilic/hydrophobic behavior of the material in an aqueous environment.^42^

The swelling degree and swelling kinetics were evaluated by the mass gain over time (Figure 6A,B). The kinetic curves show maximum mass gains before 10 min. Peaks were verified to be approximately between 90 and 270%. Barizão and associates obtained results similar to those in this study, with a maximum swelling of around 220% in starch/κ-carrageenan-based films 2.25–0.75% w/w.^19^ The short times listed to reach maximum swelling suggest a strong attraction to the aqueous medium. The results are consistent with the solubility findings, which exhibited values greater than 70%. After achieving maximum swelling, the materials begin the onset of mass loss between 5 and 10 min. Materials Pec-100, Pec-75, and Pec-50 were solubilized entirely after 10 min. Thawien and Manjeet suggest that mass loss is linked to the solubilization of compounds in solution.^43^

**Figure 6:**
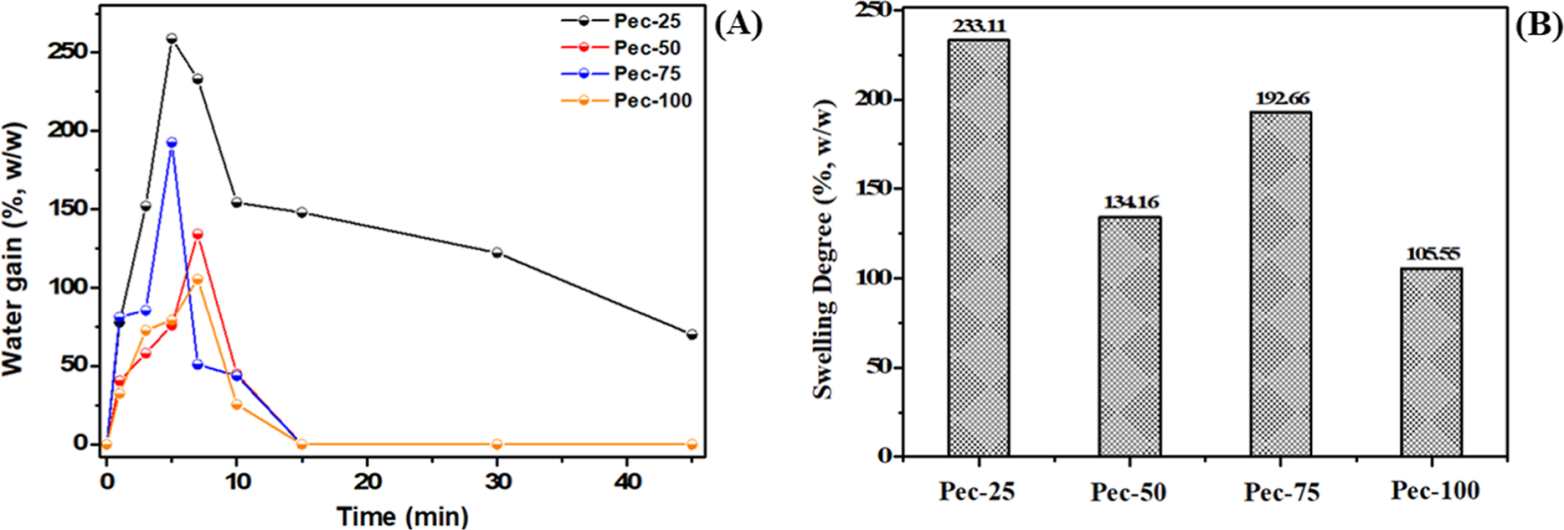
Kinetic curves (A) and swelling degree (B) of the films.

### 3.8 Color, Apparent Opacity, and UV-Blocking

Measuring the color and transparency of new package materials is essential for food applications. This measurement is directly related to the acceptance of the packaged product.^15,17^ Thus, the coloration and transparency of the new materials were determined based on apparent opacity, obtained at 550 nm, and the parameters of lightness/brightness (L*), redness/greenness (a*), yellowness/blueness (b*), color variation (Δ*E*), and whiteness index (WI) (Table 5).

**Table 5:** Color Parameters and Apparent Opacity of the Films^a^

films	L*	a*	b*	Δ*E*	WI	*A*_550_
Pec-100	88.59 ± 0.14^a^	–0.78 ± 0.02^a^	13.44 ± 0.11^a^	13.46 ± 0.03^a^	95.07 ± 0.01^a^	0.237 ± 0.03^a^
Pec-75	89.46 ± 0.48^b^	–0.71 ± 0.02^b^	11.43 ± 0.64^b^	7.1 ± 0.03^b^	94.19 ± 0.02^a^	0.205 ± 0.02^b^
Pec-50	89.91 ± 0.38^b^	–0.76 ± 0.05^b^	10.33 ± 0.54^b^	10.35 ± 0.03^a^	93.58 ± 0.01^a^	0.212 ± 0.02^b^
Pec-25	91.34 ± 0.05^c^	–0.66 ± 0.01^b^	7.07 ± 0.12^c^	11.45 ± 0.03^a^	93.31 ± 0.01^a^	0.196 ± 0.02^b^

a Results are presented as mean ± standard deviation. Different letters in the same column indicate significant differences (*p* ≤ 0.05) according to Tukey’s test. A_550_—apparent opacity.

All materials exhibited no intense colors and high transparency. The apparent opacity of the films varied from 0.196 to 0.237 (Table 5). The Pec-75, Pec-50, and Pec-25 mixtures reveal opacity measurements lower than the Pec-100 (*p* ≤ 0.05). According to Domene-López and coauthors, low absorption values indicate highly transparent materials. The authors published a comparative study on the properties of different starch-based films.^44^ Furthermore, high L* and WI values, followed by low Δ*E*, a*, and b* measures, suggest colorless materials. The digital images shown in Figure 1 confirm these findings. The Pec-75 and Pec-50 materials were similar for the L* parameter and different from Pec-100 and Pec-25 (*p* ≤ 0.05). In addition to being better accepted by consumers, transparent packaging materials may also exhibit antimicrobial activity. An earlier study has reported biodegradable starch-based films with high transparency and no colors.^45^ However, colorful materials can be produced depending on the nature of the additive added to the filmogenic blend, for example, starch films loaded with orange essential oil^46^ and inulin/Lactobacillus casei^45^ produced colorful materials.

Beyond visible light, blocking ultraviolet (UV) light by food packaging also plays a crucial role in food preservation. UV radiation incident on food can activate oxidation reactions, accelerate degradation processes, and reduce shelf life.^17^ UV radiation is classified based on wavelength into UVA (400–315 nm), UVB (315–280 nm), and UVC (280–200 nm).^47^ Figure 7 presents the transmittance spectra for pectin/starch films. All blends exhibit blocking at UVC and partial UVB up to 300 nm. However, the mixture composition differs in transmittance at 300–400 nm. Films rich in pectin demonstrate more efficient blocking in this spectrum region, showing low transmittance. The −COOH and −COOR (*R* = −CH_3_ and −C_2_H_5_) groups present in pectin contribute to this outcome because they absorb the light at this wavelength range through (*n* → π*) transitions.^24^ Pectin films (10%) show similar UV barrier behavior with effective blocking in the same spectral regions. However, incorporating zinc and silver nanoparticles enhances UV-blocking performance.^48^

**Figure 7:**
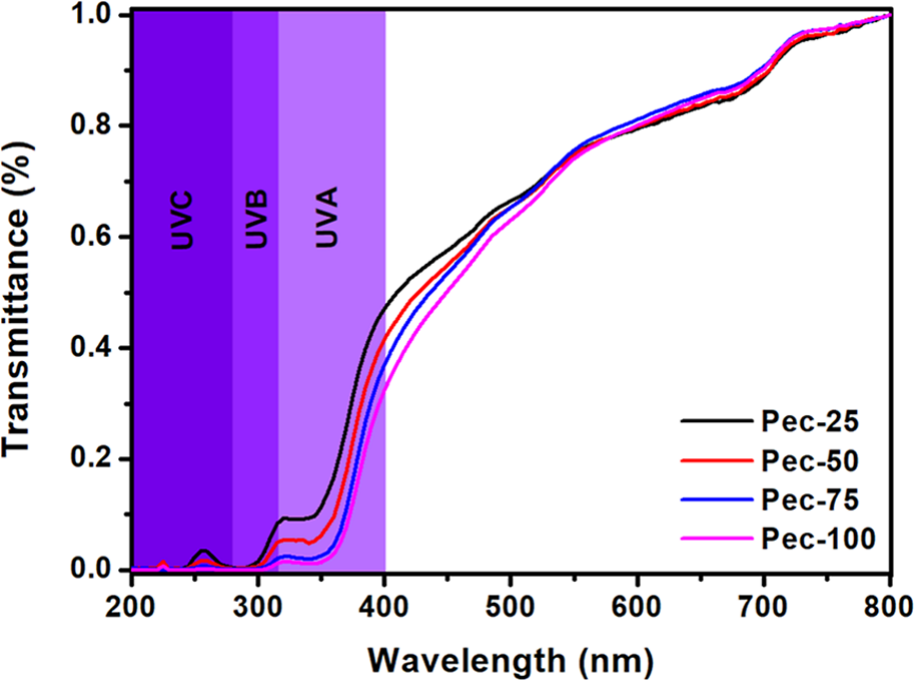
UV-blocking of the pectin/starch films.

### 3.9 Principal Component Aanalysis

Principal component analysis (PCA) assessed the correlation between materials (Pec-100, Pec-75, Pec-50, and Pec-25) and the results of mechanical property measurements (σ (MPa), ε (%), and ∑ (MPa)), WPV, swelling degree (SW %), water solubility (WS %), WI, Δ*E*, and apparent opacity (*A*_550_). The statistical analysis suggests two principal components to explain 95.31% of the total variance. The first principal component (PC1) contributed 68.27% and the second one (PC2) contributed 27.04%. Thus, PC1 has a significant influence on the total data variation.

Figure 8A depicts the projection of materials vs PC1 and PC2. Different results stemming from the measurements influenced the formation of the groups. PC1 suggests a group in the negative quadrant (Pec-100), a group in the positive quadrant (Pec-25), and another group close to the center (Pec-50 and Pec-75). The PC2 view indicated the formation of two groups, one in the positive quadrant (Pec-100 and Pec-25) and the other in the negative quadrant (Pec-75). On the other hand, Figure 8B supports the measurements responsible for forming each group. The measurements of σ, Σ, and WVP separated the Pec-100 formulation from the other films. The results show that Pec-100 exhibited high tensile strength and rigidity, contrasted with low WVP. Pec-25 showed contrary results, offering lower mechanical resistance and higher permeation to water vapor. The results are consistent because rigid materials make the vapor passage difficult. Thus, these findings support the choice of Pec-100 material in fruit film application.

**Figure 8:**
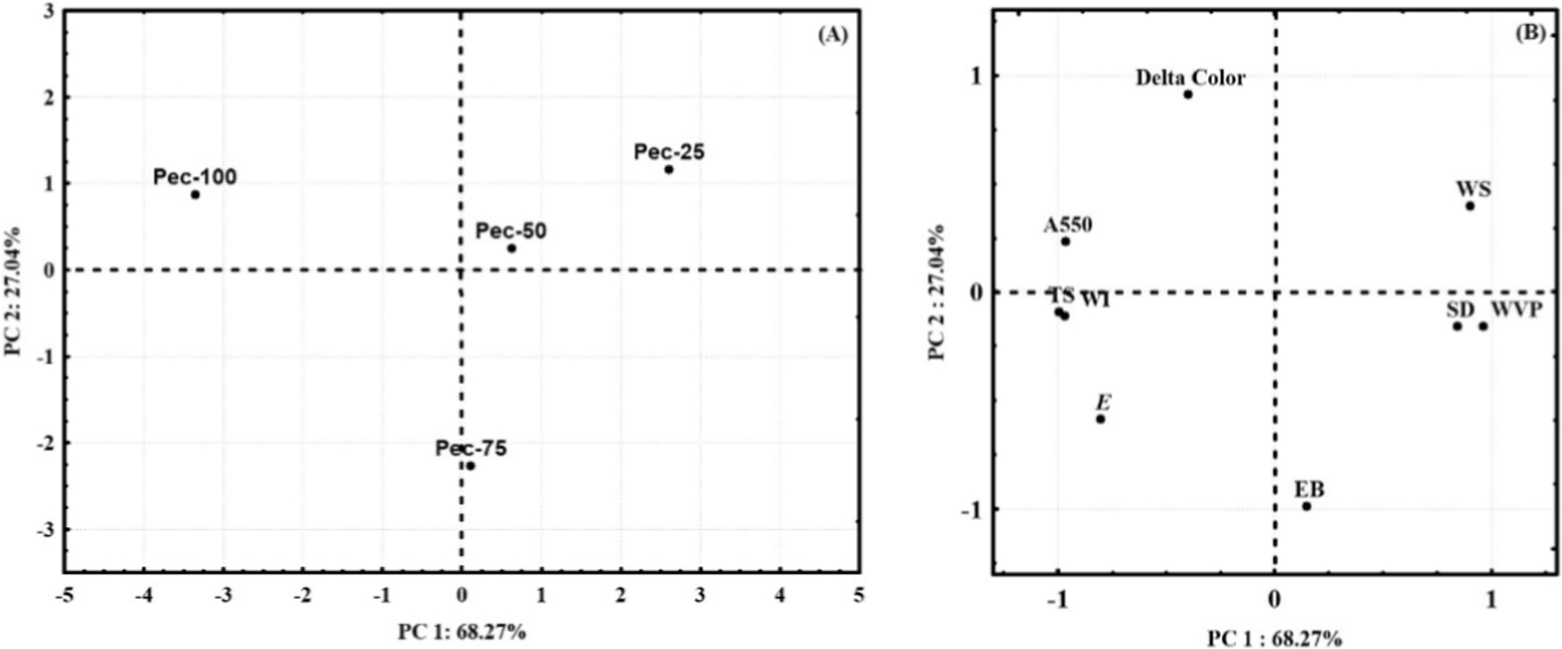
PCA of the films (A) and measurements (B). PCA: principal component analysis; σ: tensile strength; ε: elongation at break; *E*: Young’s modulus; WVP: water vapor permeation; WS: water solubility; SD: swelling degree; WI: white index; *A*_550_: apparent opacity; and ΔE: color variation.

### 3.10 Film Performance in the Preservation of Pear Fruit

The performance of the Pec-100 material applied as a film on fresh pears was evaluated over 15 days (0, 5, 10, and 15). The fruits were kept in a climate-controlled environment at 25 ± 2 °C. Figure 9 presents digital images of coated and uncoated fruits (control). Noticeably, the ripening of uncoated fruits begins on the fifth day of the experiment. A similar event was observed only on the 10th day of the experiment for the coated fruits. Additionally, brown spots (incipient rot) on the bottom of the control fruits appeared on the tenth day. The degradation points intensified on the 15th day. Conversely, the coated fruits exhibited no signs of rotting during the same testing period, showing the efficiency of the film material. The weight loss results (Figure 9B) are consistent with those of the digital images. The outcome increases throughout the storage period, reaching its highest value at the final measurement (15 days). Tukey’s test (*p* ≤ 0.05) shows significant differences in measurements between the same samples at different periods and between different samples at the same period. The coated samples provide a more effective barrier to weight loss, preserving the coated fruits longer. Thus, these findings support the possibility of applying Pec-100 as a film for fresh fruits, such as pears.

**Figure 9:**
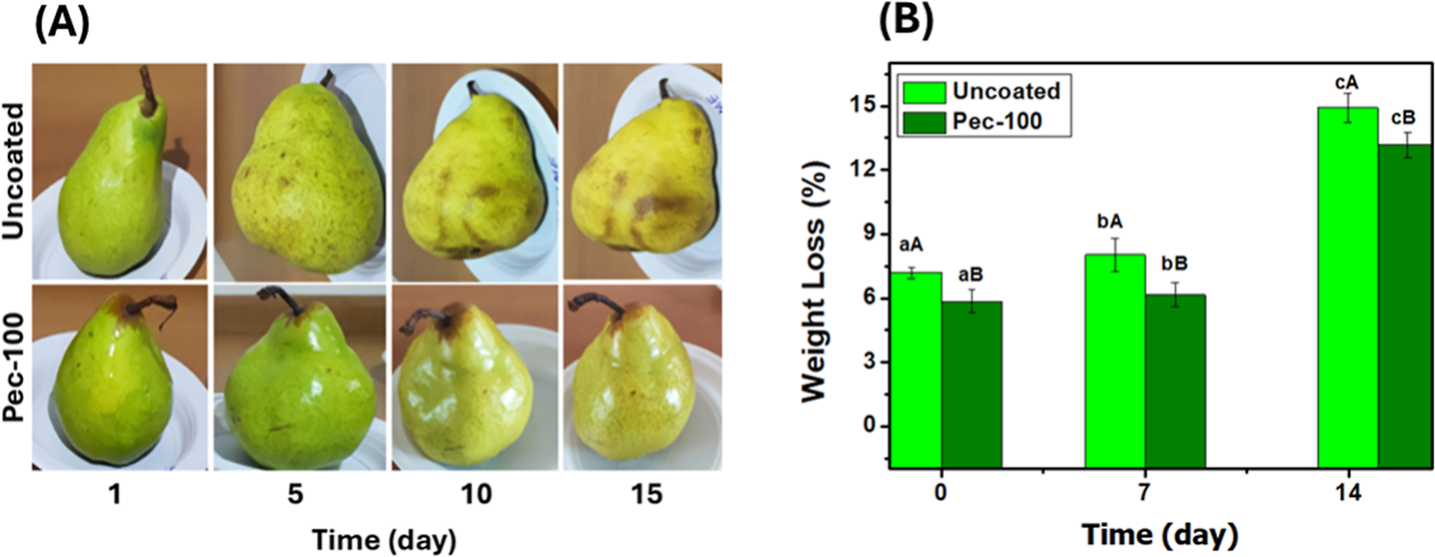
Digital images (A) and weight loss (%) (B) of the coated (Pec-100) and uncoated pears*. *The results are presented as means followed by standard deviations. Different lowercase letters indicate significant differences within the same samples at different intervals. Different uppercase letters indicate significant differences between different samples at the same period.

## 4 Conclusions

Biodegradable and UV-blocking films based on pectin/starch were produced, characterized, and applied as films on fresh pears. The results optimized via the Simplex lattice mixture design model suggest that the Pec-100 material exhibited superior tensile strength and low WVP. Additionally, PCA indicated that the material possesses transparency and rigidity, which are essential for film applications. Consequently, Pec-100 material was applied as a film on fresh pears. The results indicate that Pec-100 was effective in fruit preservation. The film maintained the fresh appearance of the coated fruits even after 15 days of storage, in contrast to the control fruits, which showed signs of degradation starting from the tenth day of the study. Therefore, the Pec-100 film material is promising for use as a film for fresh pears, effectively prolonging their shelf life.

Thus, the films were named Pec-100, Pec-75, Pec-50, Pec-25, and Pec-0. Pec-100 is 100% pectin, while Pec-0 is 100% starch. The levels of PVA (reinforcing material) and glycerol (plasticizer) were kept constant. The preparation steps are summarized in Scheme 1. First, PVA and glycerol were dissolved in distilled water over magnetic stirring at 500 rpm. Next, different portions of pectin and starch were added and stirred for 20 min. Afterward, the resulting mixture was heated at 80 ± 2 °C until complete solubilization. Subsequently, bubbles inside the solution were removed using an ultrasonic bath for 10 min (ELMA, Elmasonic P, Brazil). Then, the deaerated solutions were placed in Petri dishes (150 × 15 mm) and transferred to oven at 40 ± 2 °C for 48 h. The dried films were stored in parchment paper and conditioned in a vacuum atmosphere (desiccator).
